# Explainable Knowledge-Guided Algorithm for Contrast Extravasation Detection on Computed Tomography

**DOI:** 10.1109/JTEHM.2026.3681662

**Published:** 2026-04-07

**Authors:** Tuan D. Pham, Maki Kitamura, Taichiro Tsunoyama

**Affiliations:** Barts and The London School of Medicine and DentistryQueen Mary University of London 4617 E1 2AD London U.K.; Tokyo Metropolitan Bokutoh Hospital Sumida-ku Tokyo 130-8575 Japan; School of MedicineTeikyo University 13094 Tokyo 173-8605 Japan

**Keywords:** Trauma, contrast media extravasation, computed tomography, clinical knowledge, knowledge-guided detection.

## Abstract

Objective: To develop an explainable, knowledge-guided framework for automated detection of contrast media extravasation from sequential computed tomography (CT) images and to evaluate its potential to accelerate time-critical trauma triage while maintaining clinically acceptable sensitivity.Methods: A mathematical framework was formulated to explicitly encode three expert-derived diagnostic rules: 1) progressive increase of contrast outside anatomically plausible vessels, 2) appearance of contrast in non-vascular regions, and 3) localized irregularity of vessel caliber. Sequential two-dimensional CT slices were analyzed using a 2.5D formulation integrating temporal intensity evolution, anatomical plausibility, vessel morphology, and inter-slice continuity. The model outputs a confidence score and a binary alert. Model parameters and decision thresholds were initialized using a single representative clinical case guided by expert interpretation. Performance was evaluated against senior emergency surgeon assessment, emphasizing sensitivity and time-to-decision.Results: The proposed framework achieved clinically acceptable sensitivity for detection of contrast extravasation while substantially reducing time-to-decision relative to manual review. Early-trigger analysis demonstrated that positive cases were identified within the initial portion of the CT volume, supporting rapid screening and prioritization in emergency workflows.Conclusion: This study demonstrates the feasibility of translating expert clinical reasoning into an interpretable computational model for time-critical imaging tasks. The knowledge-guided design enables rapid automated screening while preserving transparency and clinician oversight. The framework shows promise as a decision-support tool for accelerating trauma triage, with future work focused on prospective validation and broader multi-center evaluation. Clinical Impact: The proposed knowledge-guided algorithm enables rapid extravasation alerts on trauma CT, supporting earlier triage and prioritization for angiography or surgery within existing emergency imaging workflows.

## I. Introduction

Traumatic hemorrhage remains a leading cause of preventable mortality worldwide, particularly in the early phase following injury when rapid diagnosis and timely intervention are critical [1], [2], [3]. In emergency trauma care, contrast-enhanced computed tomography (CT) plays a central role in identifying active bleeding, guiding triage decisions, and determining the need for angiographic embolization or surgical intervention [4], [5]. One of the most important radiological signs of active hemorrhage is contrast media extravasation [6], [7], [8], [9], which manifests as progressive hyperattenuation outside vascular structures across sequential CT slices or between contrast phases.

Despite advances in imaging technology, detection of contrast extravasation remains heavily dependent on expert visual interpretation [7], [10], [11], [12]. Emergency surgeons and radiologists must manually inspect CT data, scrolling through contiguous slices and comparing temporal phases under significant time pressure. Subtle leakage, complex anatomy, motion artifacts, and concurrent traumatic injuries can further complicate interpretation. In high-acuity environments, delayed identification of extravasation may postpone definitive hemostatic intervention and adversely impact clinical outcomes [13], [14].

Recent developments in artificial intelligence (AI) have demonstrated promising performance across many medical imaging applications. However, many existing approaches rely on large annotated datasets and opaque deep learning models that may limit interpretability, reproducibility, and clinical trust. Within trauma imaging, prior AI studies have largely focused on related but indirect tasks such as detection of solid organ injury [15], [16], hemorrhage surrogates [17], [18], or post-interventional contrast differentiation [19], [20], rather than explicit identification of active contrast extravasation on sequential CT series. Computational frameworks that formally encode expert diagnostic reasoning for extravasation detection and explicitly consider time-to-decision in a triage context remain limited in the current literature.

In clinical practice, experienced surgeons rely on a small number of well-defined diagnostic principles when identifying contrast extravasation [21], [22], including progressive increase of contrast outside anatomically plausible vessels and localized irregularity of vessel caliber near the suspected bleeding site. These rules are applied sequentially and contextually rather than on isolated images, reflecting a 2.5D reasoning process that integrates spatial continuity and temporal evolution.

Motivated by this observation, a knowledge-guided mathematical framework is developed that explicitly encodes expert diagnostic rules into a computational model for automated extravasation detection. The proposed approach operates on sequential two-dimensional CT slices, integrates temporal intensity evolution, anatomical plausibility, vessel morphology, and inter-slice continuity, and produces both a confidence score and a binary alert in a computationally efficient manner. This design preserves clinical interpretability while supporting integration into time-critical trauma workflows.

Unlike data-intensive deep learning approaches, the proposed framework is designed to require only minimal parameter calibration. In this study, a single representative clinical case was used to initialize model parameters and decision thresholds, guided by expert interpretation. This initialization strategy reflects a practical low-data setting and illustrates a deployment scenario in which large annotated datasets are unavailable. Furthermore, while prior studies have explored automated hemorrhage detection, the proposed work differs in three key aspects: (1) it focuses specifically on contrast extravasation (active bleeding) rather than general hemorrhage, (2) it uses a knowledge-guided, explainable mathematical framework derived from expert diagnostic rules rather than data-driven learning, and (3) it emphasizes rapid time-to-decision for emergency triage.

The primary contributions of this work are threefold: (1) formulation of a clinically explainable mathematical model that emulates expert reasoning for extravasation detection; (2) development of a computationally efficient framework for automated analysis of sequential CT data; and (3) evaluation against expert surgeon assessment with emphasis on sensitivity and time-to-decision as clinically relevant endpoints. By bridging domain knowledge and automated analysis, this work advances the translation of a trustworthy mathematical framework into emergency imaging workflows.

## II. Methods

### A. Patient Data

This study included 70 patients who presented to the emergency department of Teikyo University Hospital, and Fujieda Municipal General Hospital (both in Tokyo, Japan) following acute traumatic injury and underwent contrast-enhanced CT as part of routine clinical evaluation. The cohort consisted of adult and pediatric patients with blunt trauma mechanisms, including road traffic or train accidents, falls, sporting injuries, and assaults, who were clinically assessed to be at risk of internal hemorrhage. Table 1 summarizes the patient characteristics.

**TABLE 1:** Patient Characteristics of the Study Cohort

Characteristic	Value
Number of patients	70
Sex (male/female)	53/17
Age (years)	63.27 ± 24.68
	Range: 8–95
Mechanism of injury	
Road traffic or train accident	24
Fall from height/standing	43
Sports-related injury	1
Assault	1
Crush injury	1
Primary injury region	
Head/Face	3
Thorax	8
Abdomen/Pelvis	60
Extremities (arm and leg injuries)	5
Blunt trauma	70 (100%)

The study cohort consisted of patients with clinically confirmed contrast extravasation identified on contrast-enhanced CT. Cases were retrospectively selected based on the presence of extravasation; therefore, extravasation-negative cases were not included in the current cohort. As a result, the dataset was not balanced between positive and negative cases. This study design enabled evaluation of detection sensitivity and time-to-decision, while case-level specificity could not be assessed. To address this limitation, a slice-level specificity analysis using CT slices without visible extravasation was performed.

CT examinations comprised arterial and venous phases, and in selected cases delayed-phase acquisitions were obtained to assess for ongoing bleeding. The imaging data therefore reflected real-world clinical heterogeneity in acquisition timing, contrast dynamics, and anatomical coverage, consistent with emergency trauma practice.

CT data were analyzed as sequential two-dimensional axial slices ordered anatomically along the cranio-caudal axis. The number of slices per examination varied across patients according to scan length and acquisition parameters. Image intensities were stored in standard clinical CT format and used without retrospective modification, ensuring that the dataset reflected routine diagnostic image quality.

The reference standard for contrast extravasation was determined retrospectively based on expert assessment by senior emergency surgeons, informed by CT findings, angiographic confirmation, operative reports, and documented clinical outcomes. Patients were categorized according to the presence or absence of active contrast extravasation at the time of imaging. These expert determinations served as the ground truth labels for evaluating the proposed automated detection framework.

The presence of contrast extravasation in the study cohort was confirmed by angiographic findings, which served as the clinical reference standard. Experienced trauma surgeons reviewed the CT images to localize suspected extravasation regions, while angiography provided confirmation of active bleeding. Accordingly, the reported sensitivity of the proposed knowledge-guided algorithm reflects detection of angiographically confirmed extravasation rather than reliance solely on surgeon interpretation. This approach reduces the likelihood of missed minor bleeding and provides a clinically meaningful reference for evaluating algorithm performance.

All patient data were fully anonymized prior to analysis. This study was approved by the Research Ethics Committee of Queen Mary University of London (QME25.0836).

### B. Mathematical Framework for Knowledge-Guided Detection of Contrast Extravasation

Contrast extravasation on trauma CT represents active hemorrhage in which contrast material escapes from the vasculature into surrounding tissues. Experienced emergency surgeons and radiologists identify this phenomenon by reviewing sequential CT slices, confirming spatial continuity and progressive temporal increase of a high-attenuation focus.

To emulate this clinically-grounded reasoning process, a mathematical framework is developed that explicitly models three primary diagnostic rules derived from human expert knowledge:

1) *Extravasation is identified by progressive increase in contrast attenuation or spatial extent across sequential CT slices.*
2) *Extravasation is identified when contrast appears in regions outside anatomically plausible vascular structures.*
3) *Vessel caliber (i.e., lumen diameter) is irregular in the vicinity of the suspected bleeding site.*

Because no single slice provides sufficient evidence, the proposed algorithm integrates information from neighboring slices—a process analogous to the expert’s sequential review—effectively yielding a *2.5D* interpretation of 2D CT images. The algorithm is described as follows.

#### 1) Sequential CT Representation

Let the CT scan be a sequence of *T* ordered two-dimensional slices:

\begin{equation*} \mathcal {S} = \{ I_{t}(x, y) \mid t = 1, 2, \ldots, T \}, \tag {1}\end{equation*}

where $I_{t}(x, y)$ denotes the image intensity at pixel coordinates $(x, y)$ in slice *t*. The index *t* reflects the anatomical sequence of slices (spatial order) or, in multi-phase scans, the temporal acquisition phase.

#### 2) Vessel and Region Segmentation

A binary vessel mask $V_{t}(x, y)$ is estimated from each slice by thresholding within the range of intensity values characteristic of contrast-enhanced vessels, where vessels refer to intravascular blood-filled tubular structures (e.g., arteries and veins) that normally contain iodinated contrast material and appear as highly-attenuating, spatially connected regions on CT imaging:

\begin{align*} V_{t}(x, y) = \begin{cases} \displaystyle 1, & \text {if}~ I_{t}(x, y) \in [I_{\min }^{\text {vessel}}, I_{\max }^{\text {vessel}}], \\ \displaystyle 0, & \text {otherwise}. \end{cases} \tag {2}\end{align*}

Similarly, high-density candidate regions possibly representing contrast pooling are extracted using an upper threshold:

\begin{align*} C_{t}(x, y) = \begin{cases} \displaystyle 1, & \text {if}~ I_{t}(x, y) \geq I_{\text {thr}}, \\ \displaystyle 0, & \text {otherwise}. \end{cases} \tag {3}\end{align*}

Each connected component within $C_{t}(x, y)$ defines a region $R_{t}^{k}$, with mean intensity

\begin{equation*} \mu _{t}^{k} = \frac {1}{|R_{t}^{k}|} \sum _{(x, y) \in R_{t}^{k}} I_{t}(x, y). \tag {4}\end{equation*}

#### 3) Rule 1: Progressive Increase in Contrast Attenuation Over Time

To assess whether contrast intensity increases across adjacent slices (or between early and delayed phases), the change in mean attenuation of the same region is computed as

\begin{equation*} \Delta \mu ^{k} = \mu _{t+1}^{k} - \mu _{t}^{k}. \tag {5}\end{equation*}

An increase exceeding a defined threshold indicates progressive leakage:

\begin{align*} \mathbf {1}[\text {Increase}] = \begin{cases} \displaystyle 1, & \text {if}~ \Delta \mu ^{k} > \tau _{\text {inc}}, \\ \displaystyle 0, & \text {otherwise} \end{cases} \tag {6}\end{align*}

where $\tau _{\text {inc}}$ represents the minimum increase required to consider extravasation progression significant, and $\mathbf {1}[\cdot]$ denotes the indicator function, defined as

\begin{align*} \mathbf {1}[\text {condition}] = \begin{cases} \displaystyle 1, & \text {if the condition is true}, \\[4pt] \displaystyle 0, & \text {if the condition is false}. \end{cases} \tag {7}\end{align*}

#### 4) Rule 2: Contrast Outside Vascular Structures

The spatial context of each region is assessed relative to the vessel map:

\begin{equation*} E_{t}^{k} = \frac {1}{|R_{t}^{k}|} \sum _{(x, y) \in R_{t}^{k}} \left ({{ 1 - V_{t}(x, y) }}\right ). \tag {8}\end{equation*}

When $E_{t}^{k} \approx 1$, the candidate region is entirely outside recognized vascular structures, fulfilling the anatomical exclusion criterion for extravasation.

#### 5) Rule 3: Irregular Vessel Diameter

For each vessel segment $V_{t}^{m}$, its diameter $d_{t}^{m}$ is estimated (e.g., via medial axis computation). A normalized local diameter irregularity index is then defined as

\begin{equation*} \eta _{t}^{m} = \frac {|d_{t}^{m} - d_{t-1}^{m}|}{d_{t-1}^{m}}. \tag {9}\end{equation*}

Anomalous local dilatation or tapering is identified if $\eta _{t}^{m} > \tau _{\text {diam}}$.

#### 6) Continuity Across Sequential Slices

Domain experts confirm extravasation by verifying continuity of the hyperdense focus across neighboring slices. The overlap between regions on adjacent slices is computed as

\begin{equation*} \text {Overlap} (R_{t}^{k}, R_{t+1}^{k'}) = \frac {|R_{t}^{k} \cap R_{t+1}^{k'}|}{|R_{t}^{k} \cup R_{t+1}^{k'}|}. \tag {10}\end{equation*}

#### 7) Composite Extravasation Scoring and Indicator Function

The final likelihood of contrast extravasation for a given candidate region $R_{t}^{k}$ is computed as a weighted combination of the evidences derived from the three expert diagnostic rules and the spatial continuity between consecutive slices. This is expressed as:

\begin{align*} \Lambda _{t}^{k} & = w_{1} {\,}\mathbf {1}[\Delta \mu ^{k} > \tau _{\text {inc}}] + w_{2} {\,}E_{t}^{k} \\ & \quad + w_{3} {\,}\mathbf {1}[\eta _{t}^{m} > \tau _{\text {diam}}] + w_{4} {\,}\text {Overlap}(R_{t}^{k}, R_{t+1}^{k'}), \tag {11}\end{align*}

where

• $w_{i}$ are non-negative weights that quantify the relative contribution of each diagnostic rule to the overall likelihood.
• $\Delta \mu ^{k}$ represents the increase in mean attenuation of region $R_{t}^{k}$ between adjacent slices; the term $\mathbf {1}[\Delta \mu ^{k} > \tau _{\text {inc}}]$ activates Rule 1 (contrast escape and progressive enhancement) only when the increase exceeds the defined threshold $\tau _{\text {inc}}$.
• $E_{t}^{k}$ is a continuous measure (ranging from 0 to 1) representing the proportion of the region lying outside anatomically plausible vessels (Rule 2). Higher values of $E_{t}^{k}$ imply greater anatomical inconsistency with known vasculature.
• $\eta _{t}^{m}$ quantifies the irregularity of vessel diameter for segment $V_{t}^{m}$; the binary term $\mathbf {1}[\eta _{t}^{m} > \tau _{\text {diam}}]$ activates Rule 3 (abnormal vessel morphology) when local diameter variation exceeds the threshold $\tau _{\text {diam}}$.
• $\text {Overlap}(R_{t}^{k}, R_{t+1}^{k'})$ expresses spatial continuity of the candidate region between neighboring slices, ensuring persistence of the high-density area across the sequential CT stack.

The indicator function $\mathbf {1}[\cdot]$ serves as a binary switch that converts qualitative expert decisions (e.g., “contrast increased” or “vessel irregular”) into quantitative numerical values suitable for computational combination. Each rule contributes to the total score $\Lambda _{t}^{k}$ only when its respective condition is satisfied. In contrast, continuous terms such as $E_{t}^{k}$ and the spatial overlap contribute proportionally to their measured values.

Thus, the composite likelihood $\Lambda _{t}^{k}$ fuses discrete diagnostic confirmations with continuous anatomical evidence, emulating the reasoning process of a human expert who integrates multiple criteria—contrast progression, anatomical plausibility, and morphological irregularity—to identify true extravasation. A region is finally classified as potential extravasation when

\begin{equation*} \Lambda _{t}^{k} \geq \tau _{\text {extr}}, \tag {12}\end{equation*}

where $\tau _{\text {extr}}$ is a decision threshold determined empirically through expert-labelled training data or validation analysis.

The knowledge-guided detection workflow, integrating temporal enhancement, anatomical exclusion, and vessel morphology, is summarized in Algorithm 1.

Algorithm 1 **Knowledge-Guided Detection of Contrast Extravasation From Sequential CT Slices**
**Require:** CT slice sequence $\mathcal {S}=\{I_{t}\}_{t=1}^{T}$; thresholds $I_{\text {thr}},\tau _{\text {inc}}, \tau _{\text {diam}}, \tau _{\text {extr}}$; vessel range $[I_{\min }^{\text {vessel}}, I_{\max }^{\text {vessel}}]$; weights $w_{1},w_{2},w_{3},w_{4}$
**Ensure:** Binary extravasation masks $\{M_{t}\}_{t=1}^{T}$ and region scores $\{\Lambda _{t}^{k}\}$
Initialize $M_{t}(x,y)\gets 0$ for all *t* and pixels $(x,y)$
**for**$t=1$ to *T* **do**
$V_{t}(x,y) \gets \mathbf {1}\!\left [{{I_{t}(x,y)\in [I_{\min }^{\text {vessel}}, I_{\max }^{\text {vessel}}]}}\right]$
$C_{t}(x,y) \gets \mathbf {1}\!\left [{{I_{t}(x,y)\ge I_{\text {thr}}}}\right]$
Extract connected components $\{R_{t}^{k}\}_{k=1}^{K_{t}}$ from $C_{t}$ (8-connectivity)
**end for**
**for**$t=1$ to *T* **do**
**for**$k=1$ to $K_{t}$ **do**
$\mu _{t}^{k} \gets \frac {1}{|R_{t}^{k}|}\sum \limits _{(x,y)\in R_{t}^{k}} I_{t}(x,y)~\triangleright $Rule 1: intensity increases across slices
**if**$t< T$**then**
Select a matching region index $k'$ in slice $t+1$
$\mu _{t+1}^{k'} \gets \frac {1}{|R_{t+1}^{k'}|}\sum \limits _{(x,y)\in R_{t+1}^{k'}} I_{t+1}(x,y)$
$\Delta \mu ^{k} \gets \mu _{t+1}^{k'} - \mu _{t}^{k}$
$S_{\text {inc}}^{k} \gets \mathbf {1}\!\left [{{\Delta \mu ^{k} > \tau _{\text {inc}}}}\right]$
**else**
$S_{\text {inc}}^{k} \gets 0$
**end if**$\triangleright $Rule 2: contrast in anatomically non-vascular regions
$E_{t}^{k} \gets \frac {1}{|R_{t}^{k}|}\sum \limits _{(x,y)\in R_{t}^{k}}\left ({{1 - V_{t}(x,y)}}\right)~\triangleright $Rule 3: irregular vessel diameter near the candidate
**if**$t> 1$**then**
Estimate local vessel diameter $d_{t}^{m}$ near $R_{t}^{k}$ (e.g., via medial axis)
Estimate $d_{t-1}^{m}$ on slice $t-1$
$\eta _{t}^{m} \gets \frac {|d_{t}^{m} - d_{t-1}^{m}|}{d_{t-1}^{m}}$
$S_{\text {diam}}^{m} \gets \mathbf {1}\!\left [{{\eta _{t}^{m} > \tau _{\text {diam}}}}\right]$
**else**
$S_{\text {diam}}^{m} \gets 0$
**end if**$\triangleright $Continuity across sequential slices
**if**$t< T$**then**
Let $R_{t+1}^{k"}$ be the matched region in slice $t+1$
$\text {Overlap} \gets \frac {|R_{t}^{k} \cap R_{t+1}^{k"}|}{|R_{t}^{k} \cup R_{t+1}^{k"}|}$
**else**
$\text {Overlap} \gets 0$
**end if**$\triangleright $Composite likelihood
$\Lambda _{t}^{k} \gets w_{1} S_{\text {inc}}^{k} + w_{2} E_{t}^{k} + w_{3} S_{\text {diam}}^{m} + w_{4}{\,}\text {Overlap}~\triangleright $Decision
**if**$\Lambda _{t}^{k} \ge \tau _{\text {extr}}$**then**
Set $M_{t}(x,y)\gets 1$ for all $(x,y)\in R_{t}^{k}$
**end if**
**end for**
**end for**
**return**$\{M_{t}\}_{t=1}^{T}$ and $\{\Lambda _{t}^{k}\}$

The decision to alert extravasation is made as follows:

\begin{equation*} \lambda _{t} = \frac {1}{|\mathcal {K}_{t}|} \sum _{k \in \mathcal {K}_{t}} \Lambda _{t}^{k}, \tag {13}\end{equation*}

where $\mathcal {K}_{t}$ denotes the set of candidate regions on slice *t*. If $|\mathcal {K}_{t}| = 0$, $\lambda _{t}$ is defined as zero.

\begin{align*} \lambda _{\max } & = \max _{t \in \{1,\ldots,T\}} \lambda _{t}, \tag {14}\\ P_{\text {extr}} & = \frac {1}{1 + \exp \!\left ({{-\alpha \left ({{\lambda _{\max } - \tau _{\text {extr}}}}\right )}}\right )}, \tag {15}\end{align*}

in which the parameter $\alpha $ controls the slope of the sigmoid function, and $\tau _{p}$ is the probability decision threshold.

\begin{equation*} \hat {y} = \mathbf {1}\!\left [{{P_{\text {extr}} > \tau _{p} }}\right ]. \tag {16}\end{equation*}

The binary variable $\hat {y} \in \{0,1\}$ represents the activation of an extravasation alert, where $\hat {y} = 1$ indicates a positive alert and $\hat {y} = 0$ indicates no alert.

### C. Parameter Initialization and Clinical Justification

Model parameters were initialized using clinically guided reasoning derived from expert interpretation of contrast extravasation patterns on sequential CT images. Specifically, intensity thresholds were selected to identify high-attenuation contrast-enhanced regions consistent with active bleeding, while temporal increase thresholds were chosen to reflect progressive contrast leakage across adjacent slices. Spatial continuity and vessel caliber irregularity parameters were defined to emulate expert diagnostic reasoning, where persistent hyperdense regions outside expected vascular anatomy and irregular vessel morphology suggest active extravasation.

Parameter values were initially determined from a single representative clinical scan exhibiting angiographically confirmed contrast extravasation. This knowledge-guided, one-shot initialization strategy [23], [24] was adopted to define clinically meaningful thresholds based on expert interpretation of contrast behavior across sequential CT slices. In particular, the vessel intensity range $[I_{\min }^{\text {vessel}}, I_{\max }^{\text {vessel}}]$ and candidate threshold $I_{\text {thr}}$ were selected to capture high-attenuation contrast-enhanced structures relative to surrounding soft tissue. The temporal increase threshold $\tau _{\text {inc}}$ was defined to detect progressive contrast accumulation, while the vessel caliber irregularity threshold $\tau _{\text {diam}}$ was chosen to identify abnormal vessel morphology associated with active bleeding. The composite decision threshold $\tau _{\text {extr}}$ and weights $(w_{1}, w_{2}, w_{3}, w_{4})$ were selected to balance contributions from temporal, spatial, and anatomical criteria.

These parameters were subsequently applied uniformly across all patients without further tuning or optimization. This approach emphasizes interpretability and reduces the likelihood of dataset-specific overfitting. It is acknowledged that CT acquisition parameters, including tube voltage (kVp), reconstruction kernel, and image artifacts such as beam hardening or motion, may influence intensity-based thresholds. However, the selected parameters were chosen within clinically plausible ranges and demonstrated stable detection behavior across the evaluated cohort.

Accordingly, the model parameters initialized from the representative scan were defined as follows:

$I_{\text {thr}} = 150$, $\tau _{\text {inc}} = 10$, $\tau _{\text {diam}} = 0.25$, $\tau _{\text {extr}} = 1.5$, $[I_{\min }^{\text {vessel}}, I_{\max }^{\text {vessel}}] = [{150, 230}]$, $(w_{1}, w_{2}, w_{3}, w_{4}) = (1.0, 0.8, 0.5, 0.7)$, $\alpha = 0.5$, and $\tau _{p} = 0.4$.

## III. Results

### A. Performance of the Knowledge-Guided Extravasation Detection Algorithm

Performance of the proposed knowledge-guided algorithm (KGA) was evaluated against the consensus assessment of senior emergency surgeons, which served as the clinical reference standard. Given the safety-critical nature of hemorrhage detection in trauma care, sensitivity was considered the primary diagnostic metric. In addition, time-to-decision was evaluated as a key translational endpoint. At the case level, the KGA achieved a sensitivity of 100% for detecting contrast extravasation across the evaluated cohort. No cases with angiographically confirmed active bleeding were missed.

Computational efficiency was assessed by measuring the elapsed time from loading a CT series to generation of the extravasation probability and binary decision. The average processing time computed for the KGA was 1.28 seconds per case on a standard desktop computer. In contrast, the average manual review time by emergency surgeons was 1350 seconds per case, corresponding to a mean time reduction of approximately 23 minutes per examination. The quantitative comparison is summarized in Table 2.

**TABLE 2:** Sensitivity and Time-to-Decision Comparison Between the Proposed Algorithm and Emergency Surgeons

Metric	Surgeons	KGA
Sensitivity (%)	100	100
Time-to-decision (s)	$1350~\pm ~405$	1.28 ± 1.01
Time saved per case (min)	N/A	$\approx ~23$

To characterize the sensitivity–time trade-off, an early-trigger analysis was performed by recording the earliest slice index at which the composite likelihood exceeded the detection threshold. The algorithm successfully identified about 90% of extravasation-positive cases within the first three sequential slices.

### B. Comparison With Expert Assessment and Timing Analysis

The KGA was further evaluated by direct comparison with emergency surgeon assessment in terms of detection sensitivity and time-to-decision. Timing performance was als analyzed alongside diagnostic sensitivity to assess potential clinical impact.

Sensitivity achieved by the automated system was comparable to that of expert surgeons, with both approaches correctly identifying all cases (Table 2). Importantly, the algorithm consistently produced results substantially faster than manual review. The average algorithm inference time was on the order of seconds, whereas manual interpretation required substantially longer review times due to the need for sequential slice inspection and cross-phase comparison.

Time savings achieved by automation were particularly pronounced in cases with subtle or spatially dispersed extravasation, where manual interpretation required extended scrolling and careful confirmation across neighboring slices. In several cases, the algorithm flagged suspected extravasation before a complete manual review could be completed.

**FIGURE 1.**
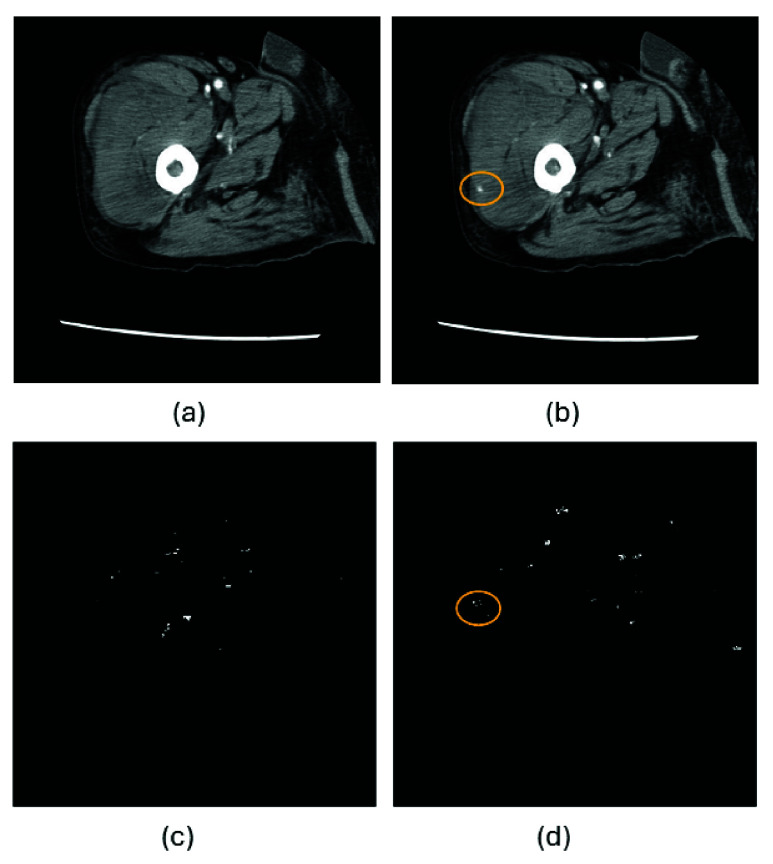
Sequential CT slices of thigh hematoma and corresponding KGA outputs for contrast extravasation detection. (a)-(b) Consecutive axial CT slices, with a surgeon-identified extravasation focus circled in (b). (c)–(d) Binary activation maps generated by the KGA, where white pixels indicate candidate extravasation regions and a positive alert is produced because the confidence score exceeds the predefined threshold. New activations in (d) correspond spatially to the suspected extravasation region.

Fig. 1 provides another illustration of contrast extravasation detection from sequential CT slices obtained from an 85-year-old male patient who sustained a thigh hematoma following a fall from standing height, together with the corresponding outputs generated by the KGA. Panels (a) and (b) show two consecutive axial CT slices acquired in sequence. In panel (b), a small focal hyperattenuating region (circled) was identified by an experienced trauma surgeon as contrast extravasation with high confidence. The finding is subtle and may be difficult to recognize rapidly during routine manual scrolling of the CT series. Panels (c) and (d) present the corresponding binary activation maps produced by the KGA, where white pixels indicate candidate regions of extravasation. The algorithm generated a positive extravasation alert with a confidence score of 0.44, exceeding the predefined detection threshold of 0.40. New clustered white activations appear in panel (d), spatially corresponding to the region highlighted by the surgeon in panel (b). This temporal emergence of localized activations across sequential slices reflects the modeled expert rule of progressive contrast accumulation and facilitates rapid visual localization of the suspected bleeding site.

**FIGURE 2.**
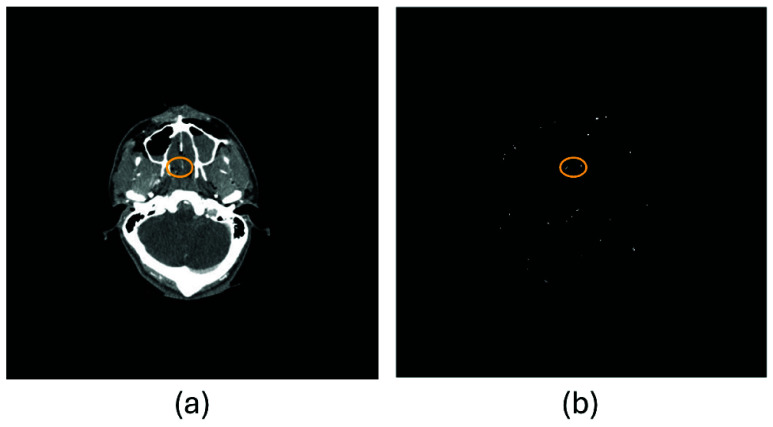
Contrast extravasation detection in a facial trauma case with fracture. (a) Axial CT slice showing a surgeon-identified extravasation focus (circled). (b) Corresponding KGA activation map highlighting candidate regions, where the extravasation region is circled and a positive alert is generated as the confidence score exceeds the detection threshold.

Fig. 2 illustrates an additional case of contrast extravasation in a 25-year-old male patient with facial trauma and fracture following a motorcycle accident. Panel (a) presents an axial CT slice in which a small focal hyperattenuating region (circled) was identified by an experienced trauma surgeon as contrast extravasation with high confidence. Panel (b) shows the corresponding binary activation map generated by the KGA, where white pixels indicate candidate extravasation regions. The algorithm produced a positive alert with a confidence score of 0.44, exceeding the detection threshold of 0.40. A localized cluster of white activations (circled) in (b) spatially corresponds to the surgeon-identified region in (a), enabling rapid visual localization of the suspected bleeding site among other candidate activations.

**FIGURE 3.**
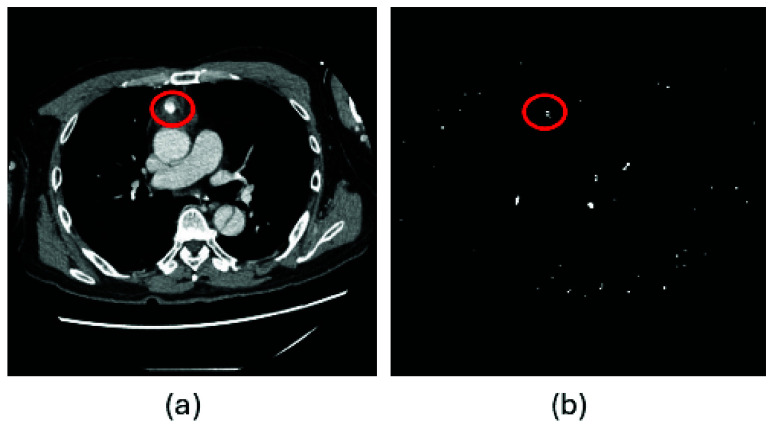
Contrast extravasation detection in a case of internal mammary artery injury. (a) Axial CT slice showing a surgeon-identified extravasation focus (circled). (b) Corresponding KGA activation map highlighting candidate regions, with the extravasation region circled and a positive alert generated.

As another illustration, Fig. 3 shows the case of contrast extravasation in a 67-year-old male patient who sustained an internal mammary artery injury following a fall from height. Panel (a) presents an axial CT slice in which a small focal region (circled) was identified by an experienced trauma surgeon as contrast extravasation with high confidence. Panel (b) shows the corresponding binary activation map generated by the KGA, where white pixels indicate candidate extravasation regions. A positive alert was generated with a confidence score of 0.44, meeting the criterion for positive detection (threshold = 0.40). The circled white activations in (b) spatially correspond to the surgeon-identified region in (a), facilitating rapid visual identification of the suspected bleeding site among other candidate activations.

### C. Perturbation Analysis of Threshold Robustness

To assess sensitivity and robustness of the proposed knowledge-guided algorithm to parameter selection, a perturbation analysis was performed by varying the candidate detection threshold $I_{\text {thr}}$ within a clinically plausible range around the baseline value. Specifically, $I_{\text {thr}}$ was varied from 140 to 160, and the resulting extravasation probability was evaluated for a representative facial trauma case as shown in Fig. 2.

**FIGURE 4.**
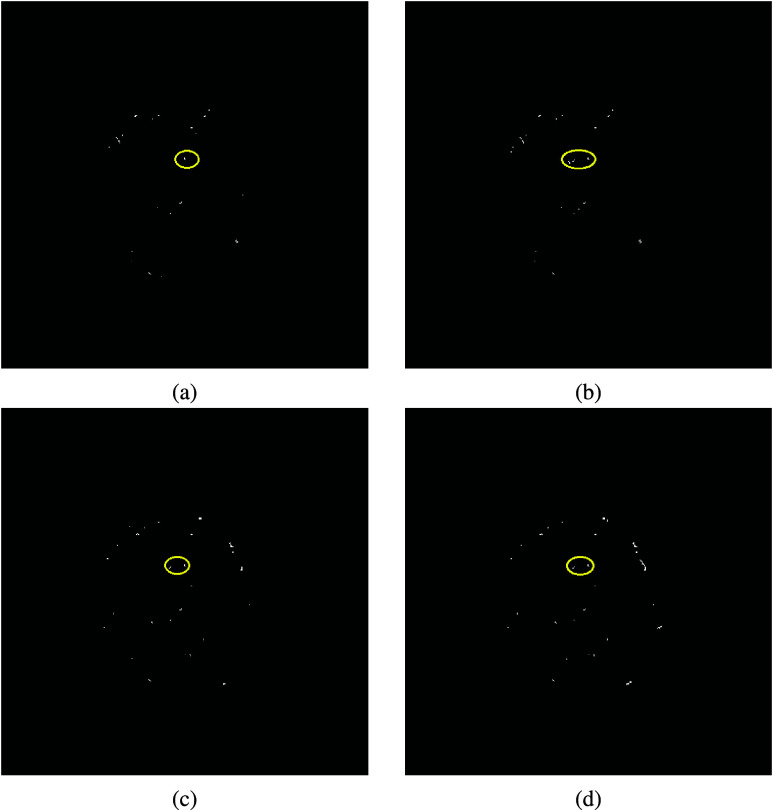
Perturbation analysis of $I_{\text {thr}}$ for the facial trauma case shown in Fig. 2. The extravasation region is circled. The confidence score (shown in brackets) exceeds the alert threshold and remains stable across threshold variations, demonstrating robustness to parameter selection: (a) 140 (0.44), (b) 145 (0.44), (c) 155 (0.43), and (d) 160 (0.43).

As shown in Fig. 4, the computed extravasation probability or confidence score remained stable across the tested range, with values between 0.43 and 0.44. Importantly, the confidence score consistently exceeded the alert threshold, resulting in unchanged positive detection across all perturbations. These findings suggest that the proposed framework is robust to small variations in threshold selection.

This perturbation analysis provides preliminary evidence that the proposed knowledge-guided algorithm maintains stable detection behavior under small parameter variations, supporting its practical applicability in real-world clinical settings.

### D. Specificity Analysis at the Slice Level

Specificity could not be assessed at the case level in the current study due to the absence of extravasation-negative cases. To provide a preliminary assessment of false-positive behavior, specificity was evaluated at the slice level using CT slices without visible extravasation from the cohort of 70 patients. Additional cropped regions of sequential CT slices without extravasation were included to increase the number of negative samples. False positives were defined as algorithm-generated alerts on these negative slices. The slice-level specificity is defined as

\begin{equation*} \text {Specificity}_{\text {slice}} = \frac {\text {TN}_{\text {slice}}}{\text {TN}_{\text {slice}} + \text {FP}_{\text {slice}}}, \tag {17}\end{equation*}

where $\text {TN}_{\text {slice}}$ and $\text {FP}_{\text {slice}}$ denote the number of true-negative and false-positive slices, respectively.

Using this formulation, the proposed method achieved a slice-level specificity of 95% (Table 3). While this analysis does not fully reflect case-level clinical decision-making, it provides a preliminary indication of the algorithm’s ability to limit false detections and complements the reported sensitivity.

**TABLE 3:** Slice-Level Specificity Analysis of the Proposed Algorithm

Metric	Value
Negative slice sets	82
True negatives ($\text {TN}_{\text {slice}}$)	78
False positives ($\text {FP}_{\text {slice}}$)	4
Slice-level specificity (%)	95.12

### E. Additional Guardrail Metrics

To assess whether the observed performance improvements reflect robust algorithm behavior rather than overfitting, additional guardrail metrics were considered. Unlike data-driven learning approaches, the proposed knowledge-guided framework does not involve model training or parameter optimization across the dataset. Instead, parameters initialized from a representative case were applied uniformly across all patients, reducing the likelihood of dataset-specific overfitting.

In addition to sensitivity and time-to-decision, slice-level specificity was evaluated to quantify false-positive behavior. This complementary metric provides an additional guardrail for assessing algorithm performance beyond detection sensitivity. The slice-level specificity results, presented in subsection III-D, demonstrate that improved time-to-decision was not achieved at the expense of excessive false-positive detections.

## IV. Discussion

### A. Impact on Clinical Decision-Making and Triage

Rapid identification of active contrast extravasation is critical for timely triage and initiation of definitive hemostatic interventions in trauma care [25], [26], [27]. Delays in detection may prolong decision-making, defer angiographic embolization or surgical exploration, and potentially increase the risk of adverse clinical outcomes. The proposed automated detection framework is therefore positioned as a clinical decision-support tool aimed at accelerating early recognition rather than replacing expert judgment.

By providing an automated extravasation probability and binary alert within seconds of CT acquisition, the system enables early prioritization of cases that require urgent review or escalation. In practical deployment, positive algorithm outputs could trigger immediate notification to the trauma team, prompting earlier consideration of angiography or interventional radiology consultation, while negative outputs may support routine review without compromising safety.

The observed reduction in time-to-decision relative to manual interpretation suggests potential improvements in emergency department throughput and workflow efficiency. In high-volume trauma settings, automated pre-screening may reduce cognitive burden on clinicians by directing attention toward high-risk studies and minimizing unnecessary delays associated with exhaustive slice-by-slice review in low-risk cases.

Importantly, maintaining high sensitivity remains essential to avoid missed hemorrhage. The knowledge-guided design of the algorithm explicitly encodes clinically interpretable rules reflecting expert reasoning, supporting transparency and facilitating clinician trust. The system therefore complements human expertise by offering rapid, consistent preliminary assessment while preserving final clinical decision authority with the treating physician.

Although this study was not designed to measure downstream clinical outcomes, the demonstrated improvements in detection speed and consistency provide compelling evidence for the potential translational value of integrating knowledge-guided automation into acute trauma imaging workflows. Prospective evaluation in real-time clinical settings will be necessary to quantify impacts on intervention timing, resource utilization, and patient outcomes.

### B. Risk Mitigation and Clinical Governance

**FIGURE 5.**
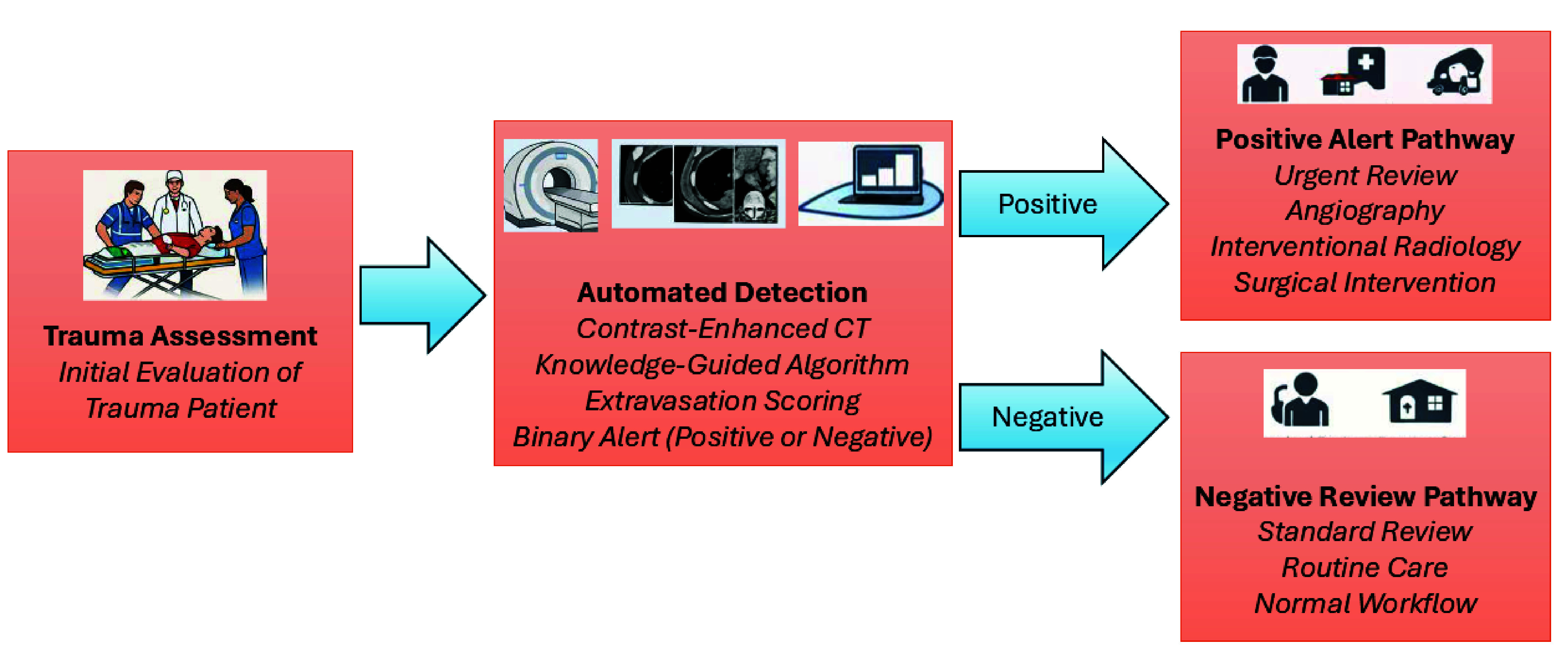
Clinical workflow integrating automated contrast extravasation detection into emergency trauma imaging: Trauma patients undergo standardized emergency assessment followed by contrast-enhanced CT imaging. The acquired CT series is automatically processed by the proposed knowledge-guided algorithm, which analyzes sequential slices to generate an extravasation score and binary alert within seconds. Positive detections trigger early prioritization and expedited review by the trauma team, supporting rapid triage and consideration of angiography, interventional radiology, or surgical intervention. Negative cases proceed through routine clinical review without workflow disruption. Final diagnostic and treatment decisions remain under clinician authority, ensuring safe integration of automated decision support into real-world emergency care.

Automated detection systems in safety-critical clinical environments must be designed with appropriate safeguards to mitigate risks associated with false alarms, algorithmic uncertainty, and unintended workflow disruption [28], [29], [30], [31]. In the proposed framework, automated outputs are intended to function exclusively as decision support rather than autonomous clinical directives (see Fig. 5). All alerts require confirmation by qualified clinicians prior to any diagnostic or therapeutic action, thereby preserving human oversight and accountability.

In healthcare, false-positive alerts may increase cognitive load or contribute to alert fatigue if not appropriately managed [32]. To mitigate this risk, the system provides a continuous extravasation probability alongside the binary alert, enabling clinicians to contextualize confidence and prioritize review accordingly. Thresholds for alert generation can be adjusted to balance sensitivity and operational burden based on local clinical requirements and institutional risk tolerance.

From a governance perspective, deployment of the system should follow existing clinical safety and regulatory frameworks, including validation on local data, prospective performance monitoring, and periodic audit of false-positive and false-negative cases. Version control, traceability of algorithm updates, and documentation of model behavior are essential to ensure reproducibility and regulatory compliance. Clear clinical ownership of deployment, escalation protocols, and incident reporting pathways should be established prior to routine clinical use.

These safeguards support safe integration of automated decision support into emergency care environments while maintaining clinician authority, patient safety, and institutional accountability.

### C. False Positives and Clinical Workflow Impact

The algorithm is designed to highlight candidate regions rather than generate definitive diagnostic conclusions. As such, isolated activations can be rapidly dismissed by clinicians when they lack continuity across sequential slices or when they occur outside anatomically plausible regions. This behavior is consistent with clinical interpretation, where experienced surgeons and radiologists evaluate persistence and anatomical plausibility across neighboring slices before confirming extravasation.

In addition, the algorithm produces results within seconds, allowing clinicians to quickly review candidate regions without introducing meaningful workflow delay. Even in the presence of multiple candidate activations, the rapid generation of alerts and spatial localization of potential bleeding sites can assist clinicians in prioritizing review of suspicious regions, thereby supporting efficient triage.

These observations indicate that while false-positive activations may occur, they are typically limited in extent and can be readily distinguished from true extravasation patterns. Consequently, the presence of false-positive candidates is unlikely to represent a significant barrier to clinical adoption, particularly given the intended role of the proposed method as an early decision-support tool for rapid screening and prioritization in trauma CT interpretation.

### D. Robustness and Overfitting Considerations

The proposed method is knowledge-guided and does not involve parameter learning from the evaluated dataset. Consequently, conventional overfitting associated with data-driven machine learning models is less applicable in this context. Parameters were initialized using clinically guided reasoning and applied consistently across all cases.

Furthermore, additional guardrail metrics, including slice-level specificity, were evaluated to assess false-positive behavior. These results support the robustness of the proposed framework and indicate that performance improvements primarily reflect workflow acceleration rather than dataset-specific optimization.

### E. Comparison With Existing Methods

Automated methods specifically designed for contrast extravasation detection in trauma CT remain limited in the current literature. Most existing studies focus on general hemorrhage detection, organ injury classification, or intracranial bleeding, which differ from contrast extravasation detection that requires sequential slice interpretation and identification of active contrast leakage.

Given this limited availability of directly comparable methods, performance comparison with expert clinician interpretation and time-to-decision was considered the most clinically relevant baseline. This comparison reflects real-world clinical practice, where rapid identification of extravasation by experienced trauma surgeons or radiologists guides urgent management decisions, including angiography, interventional radiology, or surgical intervention.

### F. Clinical Workflow Integration in Trauma Imaging

As illustrated in Fig. 5, the proposed knowledge-guided extravasation detection algorithm is designed for integration into routine trauma imaging workflows without disrupting existing clinical practice. In emergency trauma settings, contrast-enhanced CT is commonly performed following initial stabilization and clinical assessment. The acquired CT series is then reviewed sequentially by emergency surgeons and radiologists to identify active bleeding and guide urgent intervention.

In the proposed framework, the algorithm operates automatically immediately after CT acquisition. The sequential CT slices are processed in the background, and an extravasation probability together with a binary alert is generated within seconds. This rapid processing enables early identification of suspected bleeding regions while clinicians continue routine image review.

When a positive alert is generated, the system highlights candidate regions and prioritizes the case for expedited review by the trauma team. This early detection may support faster triage decisions, including consideration of angiography, interventional radiology, surgical intervention, or intensified monitoring. Conversely, when no extravasation is detected, cases proceed through routine clinical workflow without interruption.

The proposed approach functions as a clinical decision-support tool rather than a replacement for clinician judgment. Final diagnostic interpretation and treatment decisions remain under clinician authority, ensuring safe integration into emergency care environments. This workflow-oriented design supports practical translation of the proposed knowledge-guided framework into real-world trauma imaging environments, where rapid detection of contrast extravasation may improve triage efficiency and time-to-intervention.

### G. Deployment Considerations and Computational Integration

The proposed knowledge-guided extravasation detection algorithm is designed for practical deployment within existing clinical imaging infrastructures. Because the framework operates on sequential 2D CT slices and relies on computationally lightweight mathematical operations, the algorithm can be implemented without specialized hardware or high-performance computing resources.

Several deployment scenarios are feasible in real-world clinical environments. First, the algorithm may be integrated into Picture Archiving and Communication Systems (PACS), where CT images are automatically processed immediately after image reconstruction. In this configuration, extravasation probability maps and binary alerts can be generated in the background and displayed alongside standard image viewing tools, enabling clinicians to review algorithm outputs during routine interpretation.

Second, workstation-based deployment is possible, where the algorithm runs locally on radiology or trauma workstations. In this setting, sequential CT slices are automatically analyzed upon loading of the study, and candidate extravasation regions are highlighted to assist rapid identification. This approach requires minimal modification of existing clinical workflows and allows flexible implementation across institutions.

Third, background processing within clinical imaging pipelines can be implemented, where CT studies are automatically routed to a processing server immediately following acquisition. The algorithm then generates extravasation alerts and returns results to PACS or clinical dashboards. This configuration enables continuous automated monitoring without interrupting clinician workflow.

Because the algorithm requires only standard image intensity information and sequential slice processing, it is robust to typical clinical imaging environments and compatible with existing CT reconstruction pipelines. Furthermore, the short computational runtime (approximately seconds per case) supports real-time or near-real-time deployment in emergency trauma settings.

These deployment options support practical translation of the proposed knowledge-guided framework into clinical environments, enabling rapid, automated decision support while maintaining compatibility with established trauma imaging workflows.

### H. Limitations and Future Work

Several limitations should be acknowledged in the present study. First, the evaluation was conducted on a retrospective cohort from a limited number of institutions, which may restrict generalizability across scanners, imaging protocols, contrast administration practices, and patient populations. Broader multi-center validation will be necessary to assess robustness under diverse clinical conditions.

The reference standard was derived primarily from expert surgeon assessment, supplemented by angiographic and operative findings when available. Although this reflects real-world clinical practice, inter-observer variability and subjective confidence may introduce uncertainty in ground truth labeling. Future studies incorporating multi-reader consensus, blinded review, and prospective adjudication would strengthen validation rigor.

The current framework focuses on sensitivity and time-to-detection as primary performance endpoints, reflecting the safety-critical nature of trauma triage. Specificity and false-positive burden were not systematically quantified in this initial study and should be evaluated in larger cohorts to characterize operational impact, alert burden, and workflow integration. Future work will include a balanced cohort of extravasation-positive and extravasation-negative cases, as well as prospective multi-center validation, to enable comprehensive evaluation of case-level specificity, false-positive rates, and overall diagnostic performance.

A formal sensitivity analysis of model parameters was not performed in the current study due to the limited cohort and knowledge-guided initialization strategy. Future work will include systematic evaluation of parameter sensitivity and data-driven optimization to assess robustness across diverse imaging conditions and trauma presentations.

The vessel segmentation and rule-based components rely on intensity-based thresholding and simplified morphological assumptions, which may be sensitive to image noise, motion artifacts, metal implants, and atypical contrast dynamics. Integration of learned vessel probability models and adaptive parameter tuning may further improve robustness and generalization.

Future work will focus on prospective real-time deployment in emergency imaging workflows to quantify effects on time-to-intervention, resource utilization, and patient outcomes. Expansion toward fully differentiable architectures may enable end-to-end optimization while preserving clinical interpretability. Additional investigation into uncertainty estimation, calibration, and human–computer interaction will be critical for safe clinical adoption. Integration with hospital information systems and automated alerting infrastructures will also be explored to facilitate scalable deployment.

## V. Conclusion

This study presents a knowledge-guided mathematical framework for automated detection of contrast media extravasation from sequential CT images, explicitly encoding clinically interpretable diagnostic rules derived from expert practice. By integrating temporal enhancement behavior, anatomical plausibility, vessel morphology, and inter-slice continuity within a 2.5D formulation, the proposed approach emulates human reasoning while enabling computational efficiency and reproducibility. The primary contribution of this work is automation and reduction in time-to-decision rather than improved diagnostic accuracy over experienced clinicians. Accordingly, the principal clinical benefit is workflow acceleration and decision support.

Experimental evaluation demonstrates that the system achieves clinically acceptable sensitivity while substantially reducing time-to-decision compared with manual interpretation by emergency surgeons. These properties support the potential utility of the framework as a rapid screening and triage tool in time-critical trauma workflows, where early identification of active hemorrhage is essential for timely intervention.

Importantly, the transparent rule-based structure facilitates interpretability, clinical trust, and safe integration into existing workflows as decision support rather than autonomous diagnosis. While further prospective validation and broader multi-center evaluation are required, the results provide compelling evidence that knowledge-guided artificial intelligence can meaningfully augment emergency imaging workflows by improving consistency, efficiency, and clinical responsiveness.
